# Comprehensive two‐dimensional gas chromatographic platforms comparison for exhaled breath metabolites analysis

**DOI:** 10.1002/jssc.202200164

**Published:** 2022-08-02

**Authors:** Delphine Zanella, Adèle Henin, Steven Mascrez, Pierre‐Hugues Stefanuto, Flavio Antonio Franchina, Jean‐François Focant, Giorgia Purcaro

**Affiliations:** ^1^ Molecular System, Organic & Biological Analytical Chemistry Group University of Liège Liège Belgium; ^2^ Gembloux Agro‐Bio Tech University of Liège Gembloux Belgium; ^3^ Department of Chemistry, Pharmaceutical, and Agricultural Sciences University of Ferrara Ferrara Italy

**Keywords:** breath analysis, comprehensive two‐dimensional gas chromatography, volatile metabolites

## Abstract

The high potential of exhaled breath for disease diagnosis has been highlighted in numerous studies. However, exhaled breath analysis is suffering from a lack of standardized sampling and analysis procedures, impacting the robustness of inter‐laboratory results, and thus hampering proper external validation. The aim of this work was to verify compliance and validate the performance of two different comprehensive two‐dimensional gas chromatography coupled to mass spectrometry platforms in different laboratories by monitoring probe metabolites in exhaled breath following the Peppermint Initiative guidelines. An initial assessment of the exhaled breath sampling conditions was performed, selecting the most suitable sampling bag material and volume. Then, a single sampling was performed using Tedlar bags, followed by the trapping of the volatile organic compounds into thermal desorption tubes for the subsequent analysis using two different analytical platforms. The thermal desorption tubes were first analyzed by a (cryogenically modulated) comprehensive two‐dimensional gas chromatography system coupled to high‐resolution time‐of‐flight mass spectrometry. The desorption was performed in split mode and the split part was recollected in the same tube and further analyzed by a different (flow modulated) comprehensive two‐dimensional gas chromatography system with a parallel detection, specifically using a quadrupole mass spectrometer and a vacuum ultraviolet detector. Both the comprehensive two‐dimensional gas chromatography platforms enabled the longitudinal tracking of the peppermint oil metabolites in exhaled breath. The increased sensitivity of comprehensive two‐dimensional gas chromatography enabled to successfully monitor over a 6.5 h period a total of 10 target compounds, namely α‐pinene, camphene, β‐pinene, limonene, cymene, eucalyptol, menthofuran, menthone, isomenthone, and neomenthol.

**Abbreviations**
HR: high‐resolution
MSI: Metabolomics Standard Initiative
QMS: quadrupole MS
TD: thermal desorption
VOCs: volatile organic compounds
VUV: vacuum UV detector

## 1 INTRODUCTION

Using volatile organic compounds (VOCs) from human biofluids as a sign of certain diseases dates to Hippocrates (400 years BC), who described the *fetor oris* and *fetor hepaticus* as a specific bad smell caused by the putrid humors in the stomach and by liver failure [1]. With the advancement of modern instrumental techniques, several hundred VOCs have been identified in the human breath as products of metabolic processes and thus linked to the physiological conditions (e.g., health or disease) [2, 3]. Thanks to its high selectivity and sensitivity, GC coupled to MS has found an important place in contributing to the understanding of breath composition [4]. The introduction of comprehensive 2‐D GC (GC×GC) was even more impactful on the discovery and the study of novel metabolites [5, 6, 7, 8, 9], and it has gained exponential popularity since its first introduction in breathomics in 2013 [10, 11]. GC×GC–MS enhances the separation power and increases the sensitivity, allowing for minimizing coelution and increasing the number of detected VOCs in breath of almost an order of magnitude. The use of other detectors than the MS has been proposed coupled to GC×GC, among which vacuum UV detector (VUV) is a very promising one thanks to its selectivity that can provide comparable and complement information than MS, but with the perspective of miniaturization in further technological development thanks to the lack of vacuum requirements [12].

Impressive analytical advancements for the analysis of breath have been performed over the years, and many studies showing the promising and effective results obtained by breath analysis to detect different diseases [13, 14, 15, 16]. Thus, exhaled breath analysis is becoming a promising medical research area, complementing blood or urine tests for specific diseases. Indeed, breath tests are relatively easy to conduct, not invasive, and patient compliant. However, compared to blood or urine tests, the breath matrix is directly affected by the environment, by the patient diet and lifestyle, and by the sampling itself that induces variations in the exhaled breath profile. This, along with the lack of standardization in the overall process, can explain the slow progress made so far in introducing effective diagnostic tools based on breath [3]. Among the several endogenous and exogenous confounding factors that impact the breath VOCs profile, gender, age, body mass index, exercise, time of the day, diet, drugs taken, and smoking habits have been reported [17]. The sampling procedure (e.g., patient preparation, sample size/stability, sampling environment, sample storage) and the analytical method (including the sampling and instrumentation) also impact breath analysis.

The “Peppermint Initiative” was created within the Sampling and Standardization focus group of the International Association of Breath Research to address these critical points of standardization and comparison of analytical methods for exhaled breath analysis [18, 19, 20]. It is inspired by the Metabolomics Standard Initiative (MSI) [21, 22], which provides comprehensive guidelines on sampling, storage, analysis, and data reporting. Nevertheless, the Peppermint Initiative is still in the preliminary phase, aiming mainly to evaluate the many techniques employed in the field of breath analysis [19, 23]. All the participating laboratories have to follow an identical standardized workflow: sampling the exhaled breath of different individuals at different given time points after the ingestion of a peppermint oil capsule provided by the “Peppermint Consortium” to assess standardization. Afterward, the exhaled breath samples are collected at precise time points and analyzed by the available analytical platforms. Signals obtained from a series of selected targeted compounds are evaluated following washout curves, which represent the evolution of peppermint oil metabolites in the exhaled breath over time [19].

The aim of this work is to verify compliance with the requested performance within the Peppermint Initiative and compare the results obtained in parallel by different GC×GC platforms in two different laboratories. An initial assessment of the sampling conditions was performed, selecting the most suitable sampling bag material and volume. Then, a single sampling was performed using the previously selected sampling bag (1 L Tedlar), followed by the trapping of the VOCs into thermal desorption (TD) tubes for the analysis with two different analytical platforms. The TD tubes were first analyzed in GC×GC (cryogenically modulated) coupled to high‐resolution (HR) time‐of‐flight (TOF) MS. The desorption was performed in split mode and the split part was recollected in the same TD tube and further analyzed by a different GC×GC (flow modulated) with a parallel detection, specifically a quadrupole (Q) MS and a vacuum UV detector (VUV).

## 2 MATERIALS AND METHODS

### 2.1 Chemicals

A standard mixture of 19 components was used for the evaluation of the different sampling bags. The standard solution (28 ‐ 53 ppm in methanol) was made of the following single standards: hexane, 1‐propanol, 2‐methyl‐2‐butanol, 1‐ethyl‐3‐methyl‐cyclopentane, 2‐hexanone, *p*‐xylene, 1‐tetradecene, 1‐pentadecene (all from Merck Millipore, Bellefonte, PA, USA) and decane, undecane, 1‐octanol, nonanal, 2,6‐dimethylphenol, 2,6‐dimethylaniline, methyl caprate, methyl undecanoate, methyl laurate, dicyclohexylamine, 2‐ethylhexanoic acid, and 2,3‐butanediol from the Grob Mix (Merck Millipore). Capsules of 200 mg made of peppermint oil (Boots pharmaceuticals, UK) were provided by the Peppermint consortium.

### 2.2 Peppermint oil extraction

**FIGURE 1:**
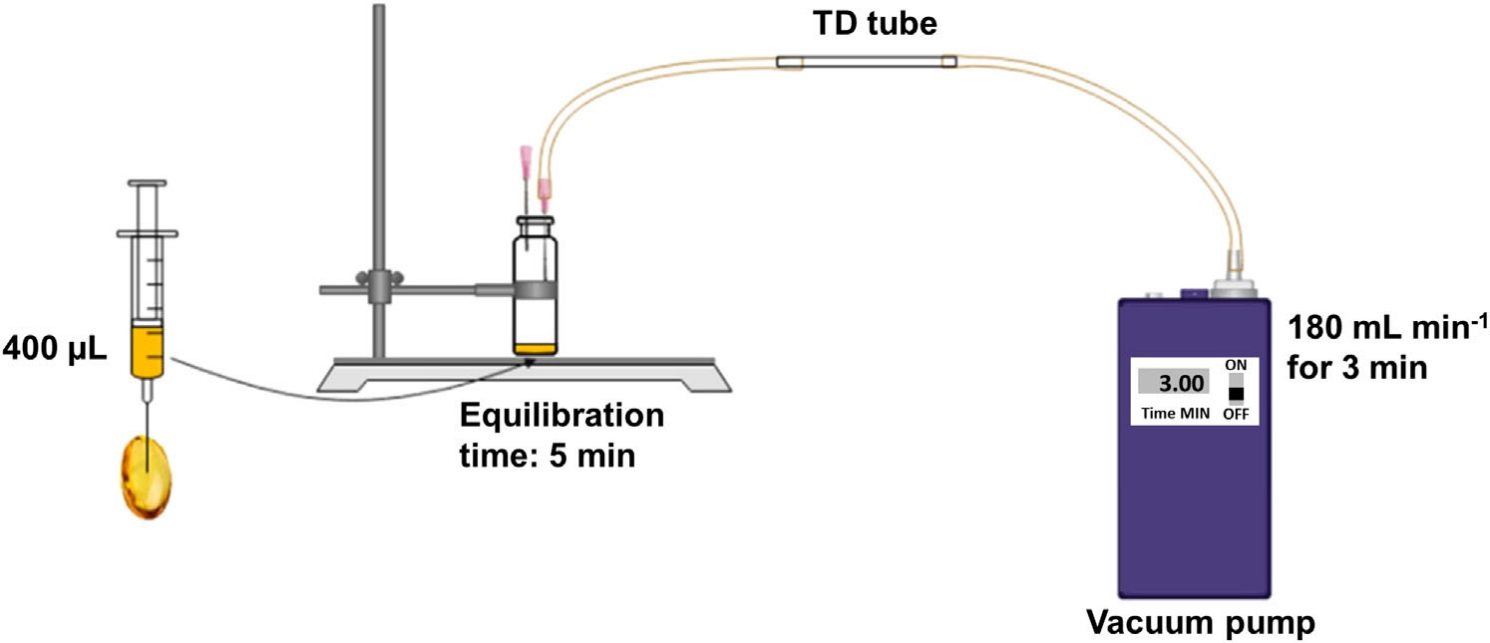
Schematic of the peppermint oil extraction from the capsule and the sampling onto thermal desorption tubes

The peppermint oil contained in one gelatin capsule, ca. 400 μL, was extracted using a glass syringe and used to select the peppermint metabolites of interest. The oil was transferred into a 20 mL headspace vial. Following an equilibration time of 5 min at room temperature (23°C), the headspace was concentrated onto a TD tube (packed with Tenax GR/Carbopack B TD) by a vacuum pump under a flow of 180 mL/min for 3 min, and further analyzed by GC×GC–TOF‐MS. A schematic of the system can be found in Figure 1.

### 2.3 Sampling optimization

Tedlar (made of a polyvinylfluoride film) and Multifoil (composed of four consecutive layers of different materials, i.e., from outer to inner layers; 60‐gauge nylon, polyethylene, 0.0003 inch aluminum foil, and 0.002 inch polyethylene) sampling bags with volumes of 1 and 5 L (Merck Millipore) were evaluated for exhaled breath collection. The content of the bags was further concentrated onto the TD tubes (packed with Tenax GR/Carbopack B TD) using a vacuum pump (ACTI‐VOC™, Markes International Ltd.), at a flow of 180 mL/min for 5 min. Initially, blank analyses of the sampling bags were carried out filling them with pure N_2_ gas to measure the extent of background contribution of the two types of material.

Furthermore, the sampling bags were evaluated using exhaled breath spiked with a standard solution. For this, each bag was initially filled (ca. 80 % of the volume) with exhaled breath from one healthy volunteer. The bag was further spiked offline with 2 μL of the standard solution (19 components) using flash vaporization at 120°C, and under a N_2_ flow of 100 mL/min for 2 min, using the adsorbent tube injector system—ATIS (Merck Millipore). All the extractions were conducted in triplicates.

### 2.4 Breath sample collection

Eight healthy participants, aged between 18 and 50 years old, were recruited for this study. Prior to and following the ingestion of a 200 mg peppermint oil capsule, exhaled breath samples were collected into 1 L inert Tedlar bags. The participants were asked to sit and to blow normally into the bag. Breath samples were collected at 7 time points; at −30 min (prior to the ingestion of the capsule), and at 0, 60, 90, 165, 285, and 360 min after the ingestion of the capsule. The exhaled breath sampling started at 8:30 am for all participants, and the samples were all collected in the same room. The participants were asked to not brush their teeth and not consume dairy products before the ingestion of the capsule. The capsule was swallowed as whole and not chewed. The Tedlar bags were concentrated onto Tenax GR/Carbopack B TD tubes (Markes International Ltd., Bridgend, UK) by means of an ACTI‐VOC vacuum pump (Markes International Ltd.), at a flow of 180 mL/min for 5.6 min. Before sampling, all Tedlar bags and TD tubes were conditioned using high‐grade N_2_, following the manufacturer's recommendations.

Sampling bags were conditioned prior to each use by filling with nitrogen up to ∼80% of their capacity and placing them in the oven at 50°C for 30 min. The bags underwent an additional UV treatment step to assure sterility, placing each bag in a Class II Biological Safety Cabinet equipped with a 255 nm UVc lamp (Thermo Scientific, Massachusetts, USA) for two 25 min cycles of sterilization. Individual single‐use joints were used to fill in the bag. As can be seen on the chromatograms of Figure S1 in the online supplement, the volatile profile of the Tedlar bag remains unchanged following the UV treatment.

The Ethics Committee of the University Hospital of Liège, reference Liege 2018/231, approved the study, and each participant signed informed consent.

### 2.5 Tube thermal desorption

Following a dry purge at 20 mL/min for 3 min, the TD tubes were desorbed at 290°C using a flow of 50 mL/min for 5 min on a general‐purpose cold trap (Markers International Ltd.) maintained at −10°C. The cold trap was then desorbed onto the GC at 300°C for 3 min using a 20:1 split for platform 1, and splitless for platform 2. Prior to and following the injection of each set of exhaled breath samples (containing 7 samples), a clean empty TD tube was injected to ensure the cleanliness of the entire system (for both analytical platforms), together with a room air sample. The sampling and analysis of room air samples allowed for the control of background contamination and more importantly, confirmed the exclusion of environmental contribution (or carryover) of the target analytes (results not reported).

### 2.6 Platform 1 (GC×GC–TOF‐MS) experimental conditions

A Pegasus GC‐HRT 4D (LECO Corporation, St. Joseph, MI, USA) equipped with an Agilent 7890 GC and a TD100‐xr thermal desorber (Markes International Ltd.) was used for the analysis. The chromatographic columns were a 30 m × 0.25 mm i.d. × 1.4 μm *d_f_* Rxi‐624SilMS as the first dimension and a 2 m × 0.25 mm i.d. × 0.5 μm d*_f_* Stabilwax as the second dimension (both from Restek Corporation). The carrier gas was helium used in constant flow mode (1 mL/min). The oven temperature program was 40°C (hold 3 min) then ramped at 5°C/min to 200°C, and finally ramped at 10°C/min to 240°C (hold 3 min). Temperature offsets for the secondary oven and for the quad‐jet dual‐stage cryogenic modulator were set at +5°C and +15°C, respectively. A 4 s modulation period was used. A mass range from 29 to 450 *m/z* was collected with an acquisition frequency of 200 Hz, in EI mode at 70 eV. An acquisition delay of 210 s was used. The transfer line and ion source temperatures were set at 220°C and 250°C, respectively.

Data were collected and analyzed using ChromaTOF^®^ HRT software version 5.20 (LECO Corporation). The signal‐to‐noise threshold was set at 100, and the NIST17 mass spectral library was used for putative identification using a spectral similarity > 70%. A standard mixture provided by the Peppermint Consortium, containing α‐pinene, β‐pinene, limonene, eucalyptol, γ‐terpinene, menthone, menthofuran, and neomenthol, was used to validate the chemical identity of these targeted compounds. Compounds resulting from the ingestion of the peppermint oil were searched using the target search function, in which the ^1^D and ^2^D retention times (tolerance of 0.2 min and 0.2 s, respectively) and the exact masses of characteristic ions (tolerance 0.01 Da) were set. The extracted raw areas were exported to Microsoft Excel for further elaboration. Instrumental blanks were performed before and after each curve to ensure that the instrument was clean, and that no carryover was present for the target analytes.

In addition, QC samples consisting in a spiked TD tube with 2 μL of a 28–53 ppm solution of exhaled breath standards, were injected weekly and enabled building a QC chart to monitor the instrumental variations (Figure S7).

### 2.7 Platform 2 (GC×GC‐quadrupole MS/Vacuum UV) experimental conditions

A Shimadzu GCMS‐TQ8050 NX (Shimadzu, Duisburg, Germany), consisting of a GC2030 coupled to a triple‐quadrupole mass spectrometer detector (TQ‐MS) (Shimadzu) and a VUV VGA‐101 Analyzer (VUV Analyzer, Texas, USA), equipped with a Centri autosampler (Markes International, Bridgend, UK) and an INSIGHT^®^ flow modulator (SepSolve, Peterborough, UK) was used.

The chromatographic columns were a 20 m × 0.18 mm i.d. × 0.18 μm *d_f_* SLB‐5 ms capillary column as the first dimension and a 5 m × 0.25 mm i.d. × 0.25 μm *d_f_* SLB‐50MS as the second dimension (both from Merck Millipore). The secondary column was then connected through an MXT Y‐union (#21389, Restek) to a 1.1 m × 0.1 mm i.d. uncoated capillary connected to the MS and 20 cm × 0.25 mm i.d. uncoated capillary connected to the VUV (flow cell dimension: 10 cm × 0.75 mm i.d.; inlet transfer line dimension: 6.1 cm × 0.25 mm i.d.). The calculated splitting flow ratio between the MS and the VUV was 41 % and 59 %, respectively.

The helium carrier gas was used in constant flow mode. The inlet pressure was programmed in order to generate a constant flow of (0.5 mL/min through the first column and 12 mL/min through the second column). The modulation time was 3.5 s with a flush pulse of 200 ms. The loop of the modulator had an internal volume of 50 μL.

The oven temperature program was the following: 40°C (hold 1 min) to 280°C (hold 4 min) at 5°C/min.

The mass spectrometer was used in EI mode at 70 eV and in scan acquisition (in single quadrupole mode) for the entire study. The scan range was set to *m/z* 35–350 with a scanning rate of 20 000 amu/s, obtaining a spectra generation frequency of 50 Hz. The MS transfer line was set to 250°C.

The transfer line and the flow cell of the VUV were set at 275°C. The VUV wavelength detection range was 125–430 nm and the data collection rate was set to 50 Hz. Nitrogen was used as the post‐column make‐up gas at a pressure of 0.63 psi.

Data were acquired by GCMS Solution (version 2.6, Shimadzu) and VUVision version 3.2.1 (VUV Analytics) and processed using ChromSpace (SepSolve Analytical). FFNSC 3.0 (Shimadzu) and NIST17s MS commercial libraries were used for identification. Putative identification was based on the combination of the MS similarity with the NIST17 library and the FFNSC library (≥80%) with the confirmation using experimental linear retention index within a ±15 range and the VUV Library. Instrumental blanks were performed before and after each curve to ensure that the instrument was clean, and that no carryover was present for the target analytes.

## 3 RESULTS AND DISCUSSION

### 3.1 Determination of the peppermint oil capsule constituents

**FIGURE 2:**
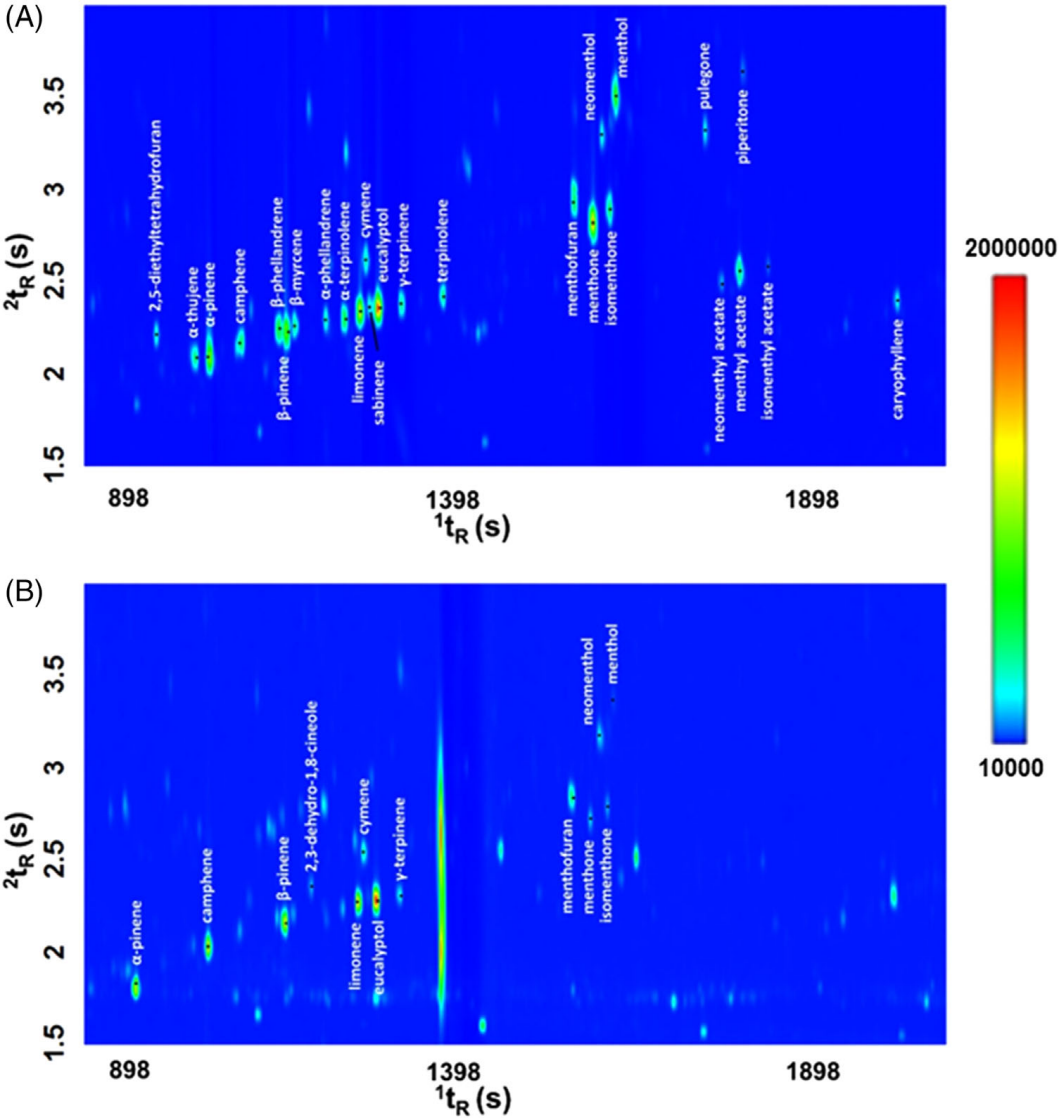
(A,B) Expansion of the GC×GC‐TOFMS 2D plot (TIC signal) of the peppermint oil headspace analysis (A), and the exhaled breath sample following the capsule ingestion (B)

The peppermint oil contained in the capsule gel provided by the Peppermint Consortium was initially analyzed by GC×GC–TOF‐MS to select the metabolites of interest. The procedure for the extraction of the oil from the capsule is described in Section 2.2. Twenty‐six major compounds were identified and are highlighted on Figure 2. Among these, 20 (i.e., 2,5‐diethyltetrahydrofuran, α‐thujene, α‐pinene, camphene, β‐phellandrene, β‐pinene, β‐myrcene, α‐phellandrene, α‐terpinene, limonene, cymene, eucalyptol, γ‐terpinene, terpinolene, menthofuran, menthone, neomenthol, pulegone, menthyl acetate, and caryophyllene) were previously identified by Malaskova et al. using classical GC–MS [24]. The remaining six identified compounds in the capsule, i.e., sabinene, isomenthone, neomenthol, piperitone, neomenthyl acetate, and isomenthyl acetate, are common constituent of the peppermint essential oil [25]. Isomenthone, neomenthol, and neomenthyl acetate are known for the minty flavor they provide to the essential oil, whereas sabinene has been characterized as fruity and spicy and piperitone as an herbaceous compound [25].

The headspace profile of the peppermint oil was compared with the profile of exhaled breath following the capsule ingestion (at *t* = 90 min). As visualized in Figure 2B, many of the volatile compounds identified in the peppermint capsule are present at very low amounts and/or even not detected in the exhaled breath of the participants.

Ten target compounds in breath were targeted in the planned temporal window: α‐pinene, camphene, β‐pinene, limonene, cymene, eucalyptol, menthofuran, menthone, isomenthone, and neomenthol.

Some compounds, like 2,3‐dehydro‐1,8‐cineole, and γ‐terpinene, were detected by only the TD‐GC×GC–TOF‐MS, but not by TD‐GC×GC–QMS/VUV. The reason for this lies in multiple factors: (I) the three detectors are inherently different, and it is well‐known that an HR TOFMS provides higher sensitivity than a QMS due to the reduced noise and so increased s/n. As well as the spectrophotometric detector (i.e., VUV) does not compare with the sensitivity of a spectrometer (i.e., MS); (II) the use of a flow modulator rather than a cryogenic one. Although more investigation would be necessary, the narrower reinjection band in the cryogenic system, along with the lower modulation number (n_M_) contribute to the higher sensitivity observed in platform 1.

These factors implicated a reduced sensitivity, thus affecting the detection of the last two compounds present at very low intensity. Indeed, 2,3‐dehydro‐1,8‐cineole and γ‐terpinene were detected in trace level in the exhaled breath of the participants using the TD‐GC×GC‐TOFMS platform. Therefore, these two compounds were treated separately, and the washout curves resulting from their analysis by TD‐GC×GC–TOF‐MS are reported in Figure S2. Once both modulator types and detectors are proved effective, a single platform (and single detector) can be used, thus avoiding any sensitivity decrease herein highlighted. However, the goal of the present study was to validate the consistency of the two platforms, which could only be achieved by analyzing the same exhaled breath samples (thus splitting them) to exclude any variation because of external factors affecting the composition such as the environment or the diet.

Table 1 reports the list of the 10 target compounds monitored with the two platforms, along with their first‐ and second‐dimension retention time, the *m/z* ions and wavelength used to quantify them in all the chromatograms for the TD‐GC×GC‐QMS/VUV platform and the TD‐ GC×GC‐TOFMS platform.

**TABLE 1:** List of the target compounds in platform 1 and 2, along with the ^1^D and ^2^D retention times and the selected fragment ions and wavelengths for quantification purposes

	Platform 1						Platform 2									
	HR TOFMS						QMS						VUV			
	^1^t_R_ (min)	^2^t_R_ (s)	*m/z*	^2^D w_b_	*n*_M_	*s/n*	^1^t_R_ (min)	^2^t_R_ (s)*	*m/z*	^2^D w_b_	n_M_	*s/n*	λ (nm)	^2^D w_b_	n_M_	*s/n*
α‐Pinene	16.14	1.72	93.0692	187	2	375	11.73	0.93	93	210	6	126	125‐160	510	6	20
Camphene	16.88	1.82	93.0693	198	3	270	12.33	1.05	93	240	5	118	125‐160	528	5	13
ß‐Pinene	17.97	1.89	93.0692	198	3	279	13.2	1.11	93	234	5	119	125‐160	546	5	20
p‐Cymene	19.83	2.33	119.0849	198	4	91	14.71	1.38	119	180	4	122	125‐160	558	4	39
Limonene	19.7	2.04	68.0614	163	3	282	14.8	1.11	68	180	6	122	125‐160	534	6	47
Eucalyptol	20.1	2.05	43.0174	165	3	350	15.09	1.3	43	240	5	116	125‐160	546	5	83
Menthone	25.3	2.58	112.0876	132	2	127	18.9	1.58	112	198	3	114	125‐160	510	3	11
Menthofuran	24.83	2.71	108.0563	237	4	131	19.08	1.64	108	240	2	120	125‐160	528	2	10
Isomenthone	25.7	2.67	112.0875	165	3	126	19.2	1.73	112	180	3	112	125‐160	492	3	18
Neomenthol	25.43	3.11	71.0485	253	3	151	19.27	1.36	71	192	3	119	125‐160	498	3	18

### 3.2 Determination of the optimal bags for exhaled breath sampling

The optimization of the sampling conditions is crucial for exhaled breath analysis to ensure reliable, repeatable, and reproducible results. The first step of exhaled breath sampling most often relies on the collection using sampling bags that are available in various materials and volumes [11, 26].

The efficiency of two different materials, i.e., Tedlar and Multifoil, and two bag volumes, i.e., 5 and 1 L, is discussed for exhaled breath analysis.

**FIGURE 3:**
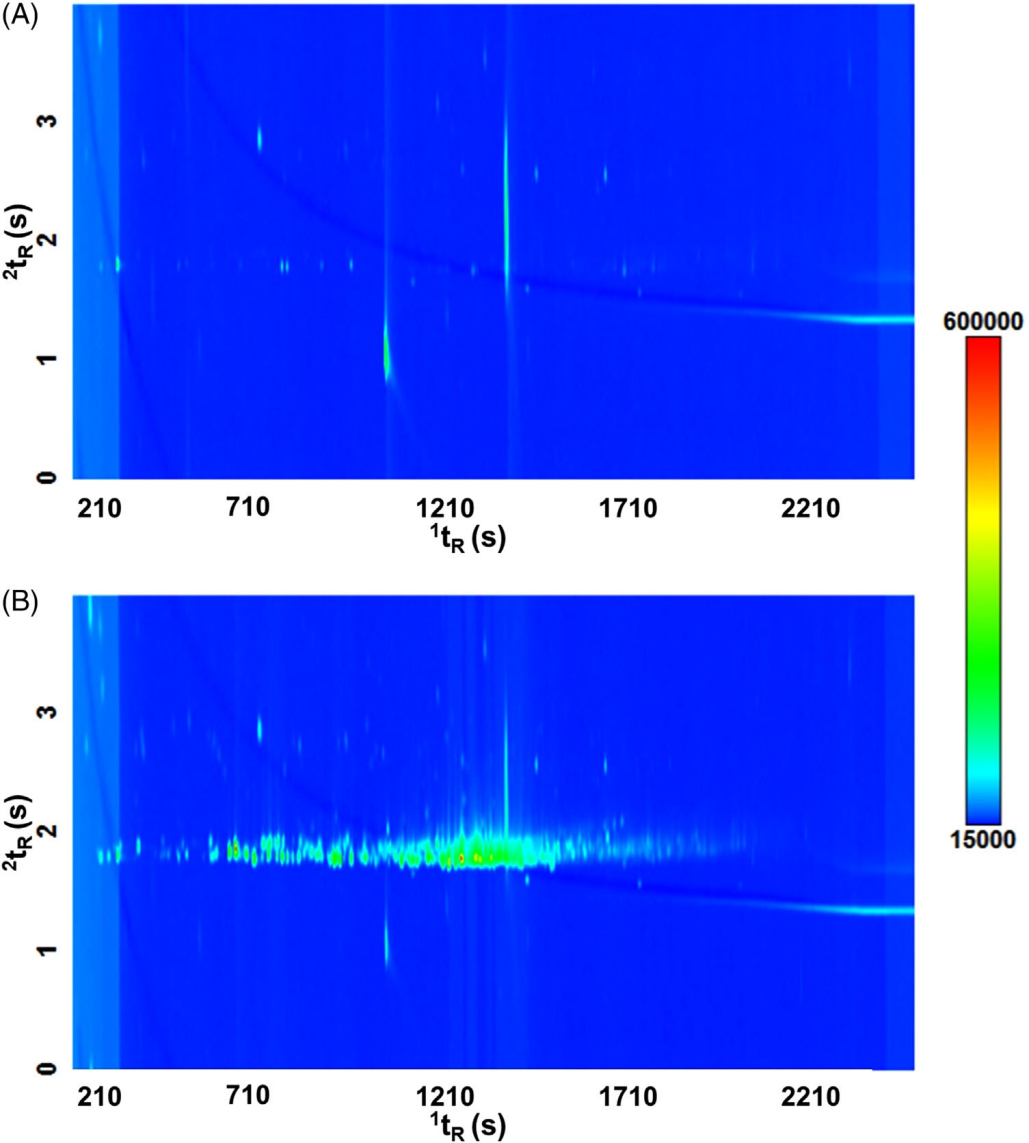
(A,B) 2D chromatograms resulting from the analysis of the background noise of A) Tedlar, and B) Multifoil sampling bags

The background contribution of the bag material was investigated using pure nitrogen, as described in Section 2.3. As can be seen on the chromatograms of Figure 3, the Multifoil background pattern is much richer in intensity than Tedlar. *N*,*N*‐dimethylacetamide, and phenol represent the two major contaminants of the Tedlar bag (Figure 3A) [27]. The Multifoil bag was characterized by an intense emission of hydrocarbons (Figure 3B), which elutes typically in a crucial area in the 2D chromatogram for exhaled breath analysis and represents a class of recurrent and important metabolites. Indeed, many hydrocarbons have been reported in previous studies as potential disease markers and linked to oxidative stress [28, 29]. This hydrocarbon signature might be explained by the composition of the Multifoil bags whose inner layer is made of polyethylene. Several bag reconditioning cycles were performed to get rid of the background noise. Nevertheless, the background composition remained unchanged following five reconditioning cycles at room temperature (Figure S3).

The evaluation of standards spiked in exhaled breath was considered to assess further the sampling capacity of the Multifoil and Tedlar sampling bags. Briefly, exhaled breath from one healthy volunteer was collected in the sampling bags which were then spiked with a standard mix using flash‐vaporization [30]. The content of the sampling bags was subsequently concentrated onto TD tubes prior to their analysis (Section 2.3).

The average response of the standards normalized by the maximum area, together with the values regarding the repeatability, are reported in Table 2. Overall, higher responses were obtained using the Tedlar bags for sampling compared to the Multifoil. Noteworthy is that the collection into Multifoil bags of low molecular weight compounds (typically associated with lower boiling points), resulted in a higher area response compared to Tedlar bags. This could be explained by the fact that Multifoil sampling bags are composed of multiple layers ensuring a low permeability for high volatile compounds.

**TABLE 2:** Response comparison of the standard compounds and repeatability using Multifoil and Tedlar sampling bags (*n* = 3). The standard compounds are ranked based on their boiling point

	Compounds	Boiling point (°C)	Vapor pressure (mmHg at 25°C)	Multifoil		Tedlar	
				Response^#^	%RSD	Response^#^	%RSD
1	Hexane	69	132	1.00	2%	0.74	1%
2	1‐propanol	98	21	1.00	5%	0.85	7%
3	2‐methyl‐2‐butanol	102	16.7	1.00	6%	0.84	4%
4	1‐ethyl‐3‐methyl‐cyclopentane	124	15.6	1.00	1%	0.87	1%
5	2‐hexanone	128	11.6	0.90	6%	1.00	1%
6	p‐xylene	139	8.9	0.46	9%	1.00	3%
7	Decane	174	1.4	1.00	15%	0.78	4%
8	1‐octanol	195	0.078	0.45	12%	1.00	9%
9	Nonanal	195	0.37	0.56	5%	1.00	5%
10	Undecane	196	0.41	Undefined*		1.00	6%
11	2,6‐dimethylphenol	203	0.27	0.43	52%	1.00	24%
12	2,6‐dimethylaniline	216	0.12	0.45	7%	1.00	13%
13	Methyl caprate	224	0.037	0.31	9%	1.00	8%
14	2‐ethylhexanoic acid	228	0.03	0.84	65%	1.00	5%
15	1‐tetradecene	233	0.015	0.85	25%	1.00	8%
16	Methyl undecanoate	247	0.027	0.25	23%	1.00	8%
17	Dicyclohexylamine	256	0.033	0.51	79%	1.00	48%
18	Methyl laurate	261	0.011	0.14	170%	1.00	6%
19	1‐pentadecene	268	0.006	0.39	11%	1.00	7%
	Average			28%		9%	
	Median (min‐max)			10% (1‐170 %)		6% (1‐48 %)	

^*^Co‐elution with the background noise of the bag.

^#^ Average area (n = 3) normalized by the highest intensity of the analyte between the two bag types.

However, the characterization of the hydrocarbons in the standard mix (i.e., decane and undecane) was hampered by the many co‐elutions with the hydrocarbon background signature of the Multifoil bag despite the HRMS deconvolution. This caused the missing integration of the undecane and the high response variability of decane (15% RSD in the Multifoil bag, against 4% RSD in the Tedlar bag), as reported in Table 2.

Sampling with Tedlar bags gave the best overall repeatability on such standards, with the average and the median of the relative standard deviation (RSD) of 9% RSD and 6% RSD, respectively. In general, the standards in the Multifoil bag were characterized by a higher and wider range of RSD, i.e., an average of 28% RSD and a median of 10% RSD (in the range of 1–170% RSD) (Table 2). The poor repeatability observed for dicyclohexylamine with both sampling bags (79% RSD for the Multifoil and 48% RSD for the Tedlar bag) can be explained by its chromatographic broadening, caused by the strong interaction with the secondary polar column (i.e., wax) resulting in lower *s/n*.

Considering (I) the low background level of the Tedlar sampling bag, (II) the overall high area responses obtained over the range of standards, and (III) the high repeatability, the following exhaled breath collections were performed using only Tedlar bags.

Although 5 L of exhaled breath are collected in many studies, reducing the sampling volume to 1 L would be beneficial for both the patients and the clinicians. Using 1 L sampling bags presents many advantages. The filling of the bags is more comfortable for the patients, especially when suffering from lung diseases. It is also time efficient for the clinician since the time required for the concentration of the exhaled breath onto the TD tube is five times shorter. Considering the time needed (per sample/patient) for transferring the breath from the bag to the TD tube, it would translate from 20 to 5 min. Nevertheless, for untargeted studies, 5 L sampling bags might be preferred to collect as many volatile metabolites as possible, and to quantitatively recollect the flow of analytes split during the cold‐trap desorption stage [31]. To prove the representativeness of the sample between the first desorption (on platform 1) and the second desorption (1 recollection, on platform 2), the efficiency of recollection was tested on the target analytes. These were considered in breath after the pill intake, comparing the first tube desorption and the following two recollection cycles (3 desorptions in total for each sample). These tests showed that the recollection efficiency (as %recovery) yielded on average 91% and 80% for the tracked metabolites (at T4, washout peak for most of the target analytes), respectively for the first and the second recollection. confirming a good representativeness of the sample in the recollected tube to be analyzed in platform 2. These results are summarized in Figure S4.

**FIGURE 4:**
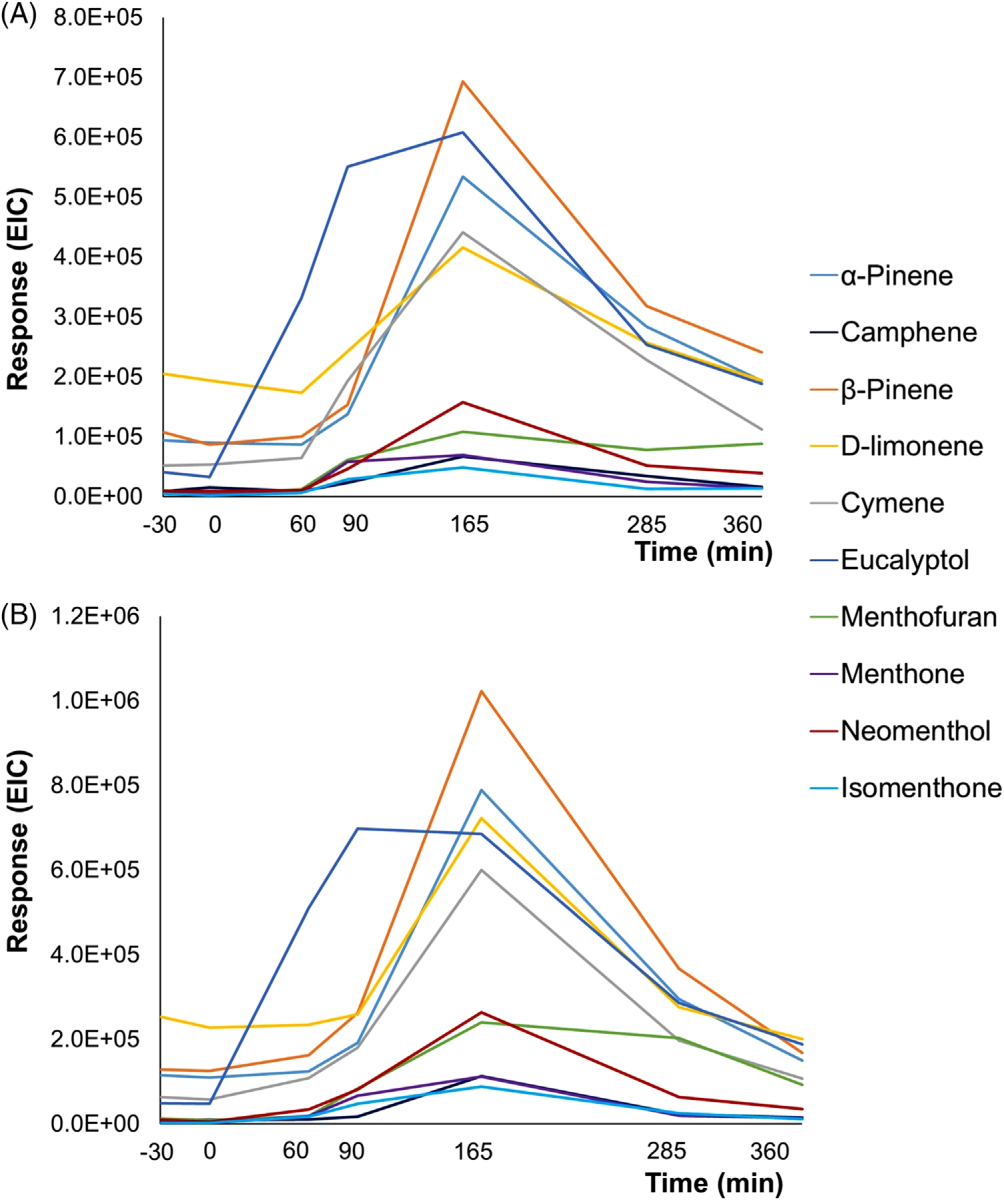
(A,B) Comparison of A) 1 L, and B) 5 L Tedlar sampling bags on metabolite intensity in longitudinal breath sampling

To further assess the feasibility of collecting less breath volume, exhaled breath samples were collected in 1 and 5 L Tedlar bags following the ingestion of a peppermint capsule. The target metabolites were then tracked longitudinally (30 min prior ingestion, 0, 60, 90, 165, 285, and 360 min). If coming from the digestion of the capsule, the expected metabolite concentration would decrease with time after a peak in intensity following its ingestion. The resulting washout curves are presented in Figure 4. As expected, the intensity of the response of the peppermint compounds decreased when using 1 L bags. However, the trend of the washout curves was similar for both 1 and 5 L sampling bags and all the targeted metabolites were detected over the defined time period. This showed the suitability of 1 L bags for exhaled breath collection and the analysis of targeted compounds in this longitudinal tracking.

### 3.3 Analytical considerations on the GC×GC platforms

The main goal of the work was to compare the agreement of the results in the context of the Peppermint Initiative and not to strictly compare the analytical performances of the two platforms or the three detectors used, for which inherent differences are well‐known, making some comparison cumbersome (e.g., the sensitivity of the three detectors). Therefore, the goal was not to reproduce the same results obtained in a GC×GC cryogenically modulated platform into a flow modulated one, as already presented by Cordero et al. [32, 33, 34], but rather to show that independently from the analytical platform used, reliable results can be obtained in the context of the Peppermint Initiative. As such, different column sets were chosen intentionally in the 2 platforms. Nevertheless, some critical steps were encountered in the optimization and some main parameters were compared.

The main difficulties were related to the optimization of the platform 2 (GC×GC‐QMS/VUV) equipped with a flow modulator, that, for its nature, is less flexible than a cryogenic one to use different settings. The goal was to have roughly equal amounts in the two detectors and to have aligned data in the two traces. all these without exceeding the maximum limit flow by the QMS and considering the high internal volume of the VUV detector (i.e., 10 cm × 0.75 mm i.d. for the flow cell plus the inlet transfer line dimension of 6.1 cm × 0.25 mm i.d.). The make‐up gas set at the VUV detector is a critical parameter that inversely affects the sensitivity (since change the residence time in the flow cell) and the peak width [12]. Higher make‐up gas would reduce the peak broadening but decrease the residence time thus the sensitivity, affecting at the same time the splitting rate and thus the alignment of the two traces and the secondary flow of the modulator. After careful calculation, a 1.1 m × 0.1 mm i.d. uncoated capillary connected to the MS and 20 cm × 0.25 mm i.d. uncoated capillary connected to the VUV (minimum length required to handle the Y‐union) were used to connect the secondary column to the two detectors. In such conditions, the flow was split between the MS and the VUV at 41 % and 59 %, respectively. The make‐up gas was fixed just slightly above the reading of the VUV flow controller when the selected secondary flow was set in order to maintain it constant all over the chromatographic run (i.e., 0.63 psi). Such a make‐up flow provided a compromise between peak width and sensitivity, although both are significantly lower than the MS performance.

Table 1 reports the comparison in terms of peak width and s/n of the targeted peaks obtained from one sample collected after 165 min from the pill ingestion. The peak widths in the flow modulated system are consistent with previously reported one, ranging between 180 and 240 ms at the MS; nevertheless, a significant band broadening caused by the dead volume of the VUV detector was observed (peak width ranging between 492 and 558 ms). The peaks were generally three times wider in the VUV than in the MS detector, which agrees with the data reported previously by Gruber *et al*. [12], who reported an increment between two and six‐time wider peaks in GC×GC‐VUV (∼300 ms) compared to GC×GC–TOF‐MS (∼180 ms). Noteworthy that in the cited study two different systems were compared without any splitting between the detectors and a cryogenic modulator was used, assuring a narrower reinjected band. Regarding the signal obtained by the two detectors (i.e., QMS and VUV) a higher *s/n* ratio was obtained for the QMS compared to the VUV (in the 1–17‐times range), considering a comparable absolute amount diverted in the two detectors.

**FIGURE 5:**
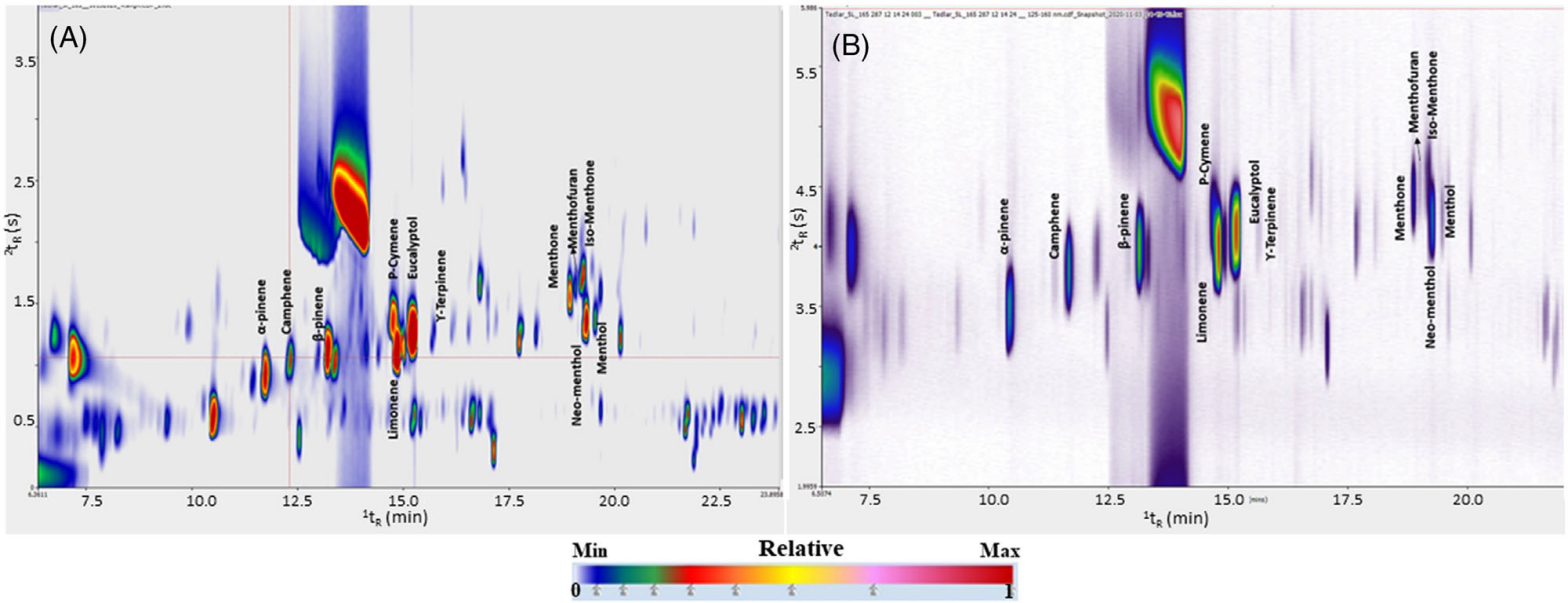
(A,B) Two‐dimensional plots obtained by TD‐GC×GC (flow modulated) coupled with the dual detection A) QMS and B) VUV

Figure 5A,B reports the two parallel chromatograms obtained with the QMS and the VUV, respectively. Because of a substantial band broadening within the VUV detector, the chromatographic resolution on this detection line was significantly impaired compared to that obtained on the MS detector line. Also, the 2D retention time between the 2 detectors is not matching; nevertheless, the 1D retention times were perfectly aligned between the two detection systems and the targeted compounds could be identified and reliably evaluated for the purpose of this study.

### 3.4 Washout curve of metabolites in exhaled breath

**FIGURE 6:**
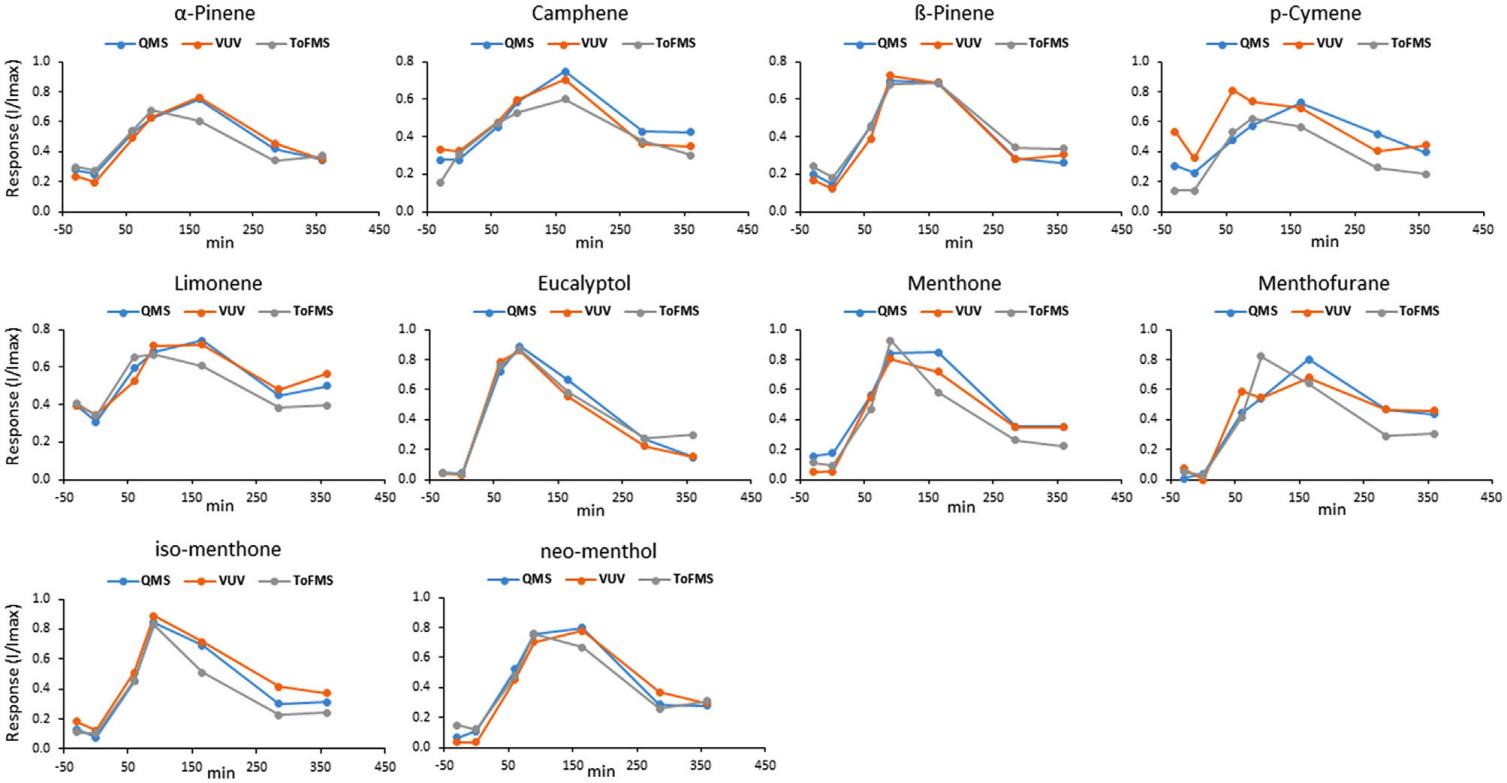
Average washout curve for the 8 participants using the TD‐GC×GC‐QMS/VUV (QMS, blue; VUV, orange), and the TD‐GC×GC‐TOFMS (TOFMS, gray) platforms

The area of each compound was normalized to compare the washout curves not only among the different volunteers (inter‐patients) but also among the two platforms and detection systems (inter‐platforms). A first normalization by the background (i.e., at time zero sampling) was performed, but because of the different sensitivity of the platforms and the low abundance of the compounds at time 0 min, the data were highly variable and not easily comparable among them. Therefore, the data were normalized by the maximum area value within each washout curve. Figure 6 reports the average results obtained with the three detector systems, while the curves with the *inter*‐patient variability are reported in Figure S5.

As seen in Figure 6, all compounds showed low or absent levels at −30 min and 0 min sampling and a clear increment after 60 min. The maximum response of the compounds is detected between 60 and 165 min, then a decreasing trend is observed (i.e., washout), generally stabilizing around 285 min, but not returning yet to the initial values obtained before ingestion of the capsule.

The washout curves obtained with the three detectors were very similar for all the target compounds, with the apex of the curve generally observed at 90 or 165 min. The response obtained across the 3 detectors for each patient taken singularly gave highly reproducible washout profiles as shown in Figure S6.

A slight difference was observed for the average curves obtained in the VUV for p‐cymene, and menthofuran (Figure 6). p‐Cymene eluted very close to limonene, but while it was well separated in GC×GC‐QMS, the broadening occurring in the VUV detector due to the high dead volume of the flow cell caused a concurrent significant loss of resolution that affected the integration of the less abundant compound (i.e., p‐cymene). Menthofuran showed a very low signal at the VUV, thus impairing the accuracy of the integration. The ideal decreasing part of the washout curve profile can be modeled with a power relationship, thus a logarithmic transformation of the descending part leads to a linear plot where the R^2^ can be evaluated to estimate the closeness to the ideal behavior [19]. Performing this exercise on our data, despite only three points were recorded after the maximum, an average *R* ^2^ (calculated as the average of the *R* ^2^ of all the compounds for each detector) of 0.938, 0.919, 0.924, for QMS, VUV, and TOFMS was obtained, respectively (Table S1). Those compounds are thus good candidates to be monitored in exhaled breath.

## 4 CONCLUSIONS

This study verified the suitability of two different GC×GC platforms and detectors in two different laboratories, in the context of the Peppermint initiative to provide a standardized instrumental approach for exhaled breath analysis. This first verification step is necessary to support the reliability of future breath studies using GC×GC (in combination with different modulator and detector types), where the full potential of this hyphenated techniques will be exploited.

A single breath sampling was performed using Tedlar bags for each individual, followed by trapping of the VOCs into TD tubes that were injected subsequently in the two laboratories. The recollection of the VOCs during the injection, which otherwise would waste the precious sample, allowed the analysis on the two platforms and the evaluation of their results.

The TD‐GC×GC‐QMS/VUV and the TD‐GC×GC–TOF‐MS platforms enabled the longitudinal tracking of peppermint oil metabolites in exhaled breath providing results in agreement. The 10 target compounds (namely α‐pinene, camphene, β‐pinene, limonene, cymene, eucalyptol, menthofuran, menthone, isomenthone, and neomenthol) were monitored successfully over a 6.5 h period. This study showed that the two platforms used are both efficient for exhaled breath analysis according to the requirement of the Peppermint initiative, allowing the longitudinal monitoring of the exhaled metabolites. The washout curves obtained with the three detectors showed good reproducibility and consistency between the two platforms. The lower sensitivity reported for the TD‐GC×GC‐QMS/VUV platform can be counterbalanced by collecting higher breath volumes, although not highly practical. The VUV can also be used as a single detector of the TD‐GC×GC platform without the need of any flow splitting as the detector can easily handle the high flow used in flow modulation, with the further advantage of reducing the analyte broadening into the detector cell. Surely the application will highly benefit from the development of reduced dead volume cell for the VUV detector and thanks to the lack of vacuum requirements a miniaturization of the technology might be a possibility in the future. This possible scenario will give significant advantage to the use of GC(×GC)–VUV in a clinical context. Further studies are necessary to prove the untargeted capability of the VUV detector, but the preliminary results published by Gruber et al., along with the verification of the performance requirements according to the Peppermint Initiative reported in this paper, set the basis for future development of the technique in the field of breath analysis.

## CREDIT AUTHOR STATEMENT

**Delphine Zanella**: Conceptualization, Methodology, Formal analysis, Visualization, Writing‐original draft, Writing‐Review & Editing; **Adèle Henin**: Formal analysis, Visualization, Investigation; **Steven Mascrez**: Formal analysis, Investigation, Methodology; **Pierre‐Hugues Stefanuto**: Conceptualization, Review & Editing; **Flavio Antonio Franchina**: Conceptualization, Methodology, Investigation, Supervision, Funding acquisition, Writing‐original draft, Writing‐Review & Editing; **Giorgia Purcaro**: Conceptualization, Methodology, Formal analysis, Visualization, Writing‐original draft, Writing‐Review & Editing, Resources, Supervision, Project Administration, Funding acquisition; **Jean‐François Focant**: Writing‐Review & Editing, Resources, Project Administration, Funding acquisition.

## Supporting information

Supplementary material
