# Supplementary material for: Comprehensive two‐dimensional gas chromatographic platforms comparison for exhaled breath metabolites analysis
Source: J Sep Sci. 2022 Aug 2;45(18):3542–55. doi: 10.1002/jssc.202200164 (PMC9804543; doi:10.1002/jssc.202200164)
Supplement: Supplementary file 1 — Supplementary material [file JSSC-45-3542-s001.docx]

**SUPPLEMENTARY MATERIAL**

**Comprehensive two-dimensional gas chromatographic platforms comparison for exhaled breath metabolites analysis**

Delphine Zanella^1,ǂ^, Adèle Henin^1,ǂ^, Steven Mascrez^2^, Pierre-Hugues Stefanuto^1^, Flavio A. Franchina^3,*^, Jean-François Focant^1,#^, Giorgia Purcaro^2,*,#^

^1^ Molecular System, Organic & Biological Analytical Chemistry Group, University of Liège, Liège, Belgium

^2^ Gembloux Agro-Bio Tech, University of Liège, Gembloux, Belgium

^3^ Department of Chemistry, Pharmaceutical, and Agricultural Sciences, University of Ferrara, Ferrara, Italy

^*^ Corresponding authors. Contact information:

Flavio A. Franchina ([flavioantonio.franchina@unife.it](mailto:flavioantonio.franchina@unife.it))

Department of Chemistry, Pharmaceutical, and Agricultural Sciences,

University of Ferrara,

L. Borsari 46,

44121 Ferrara, Italy

Giorgia Purcaro (gpurcaro@uliege.be)

Gembloux Agro-Bio Tech, University of Liège

Bât. G1 Chimie des agro-biosystèmes, Passage des Déportés 2,

5030 Gembloux, Belgium

Office phone: +32 (0)81 62 22 20

^#^ Co-last authorship

^ǂ^ Co-first authorship


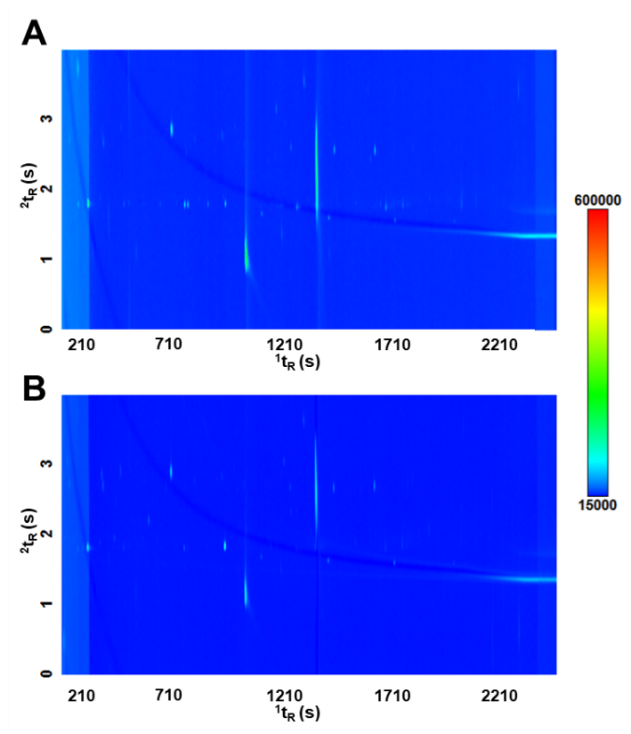


***Figure S1.*** *Chromatograms of the Tedlar bags A) prior and B) following the ultraviolet treatment for COVID-19 safety.*


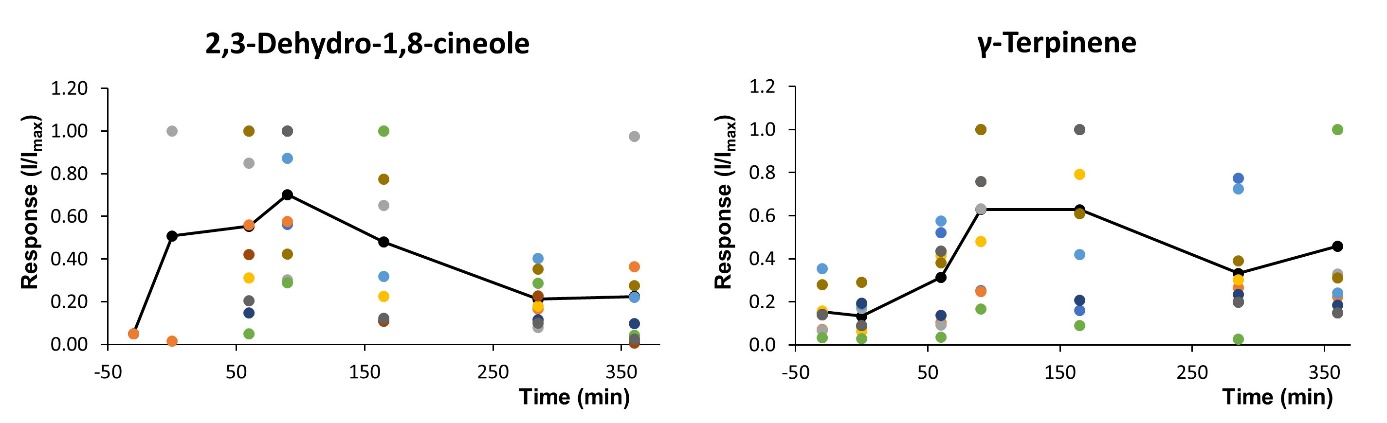


**Figure S2.** *Average washout curve for the 10 participants of 2,3-dehydro-1,8-cineole (left) and γ-terpinene (right), detected only using the TD-GC×GC-ToFMS (HRMS) (platform 1).*


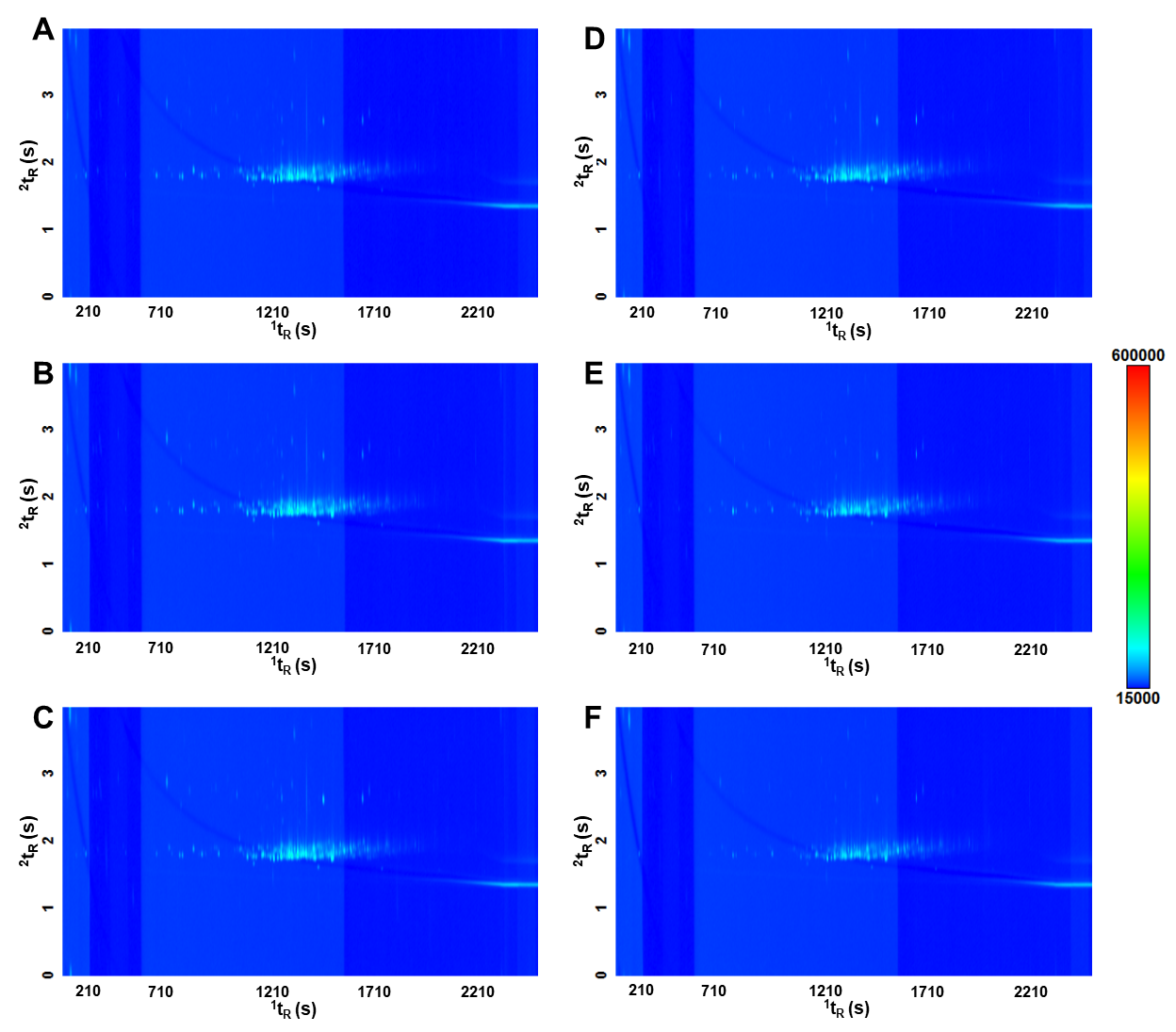


***Figure S3.*** *2D chromatograms resulting from the analysis of the background noise of Multifoil sampling bags following A-E) five reconditioning cycles, and F) following five reconditioning cycles and five fill-flush with pure N_2_.*

**
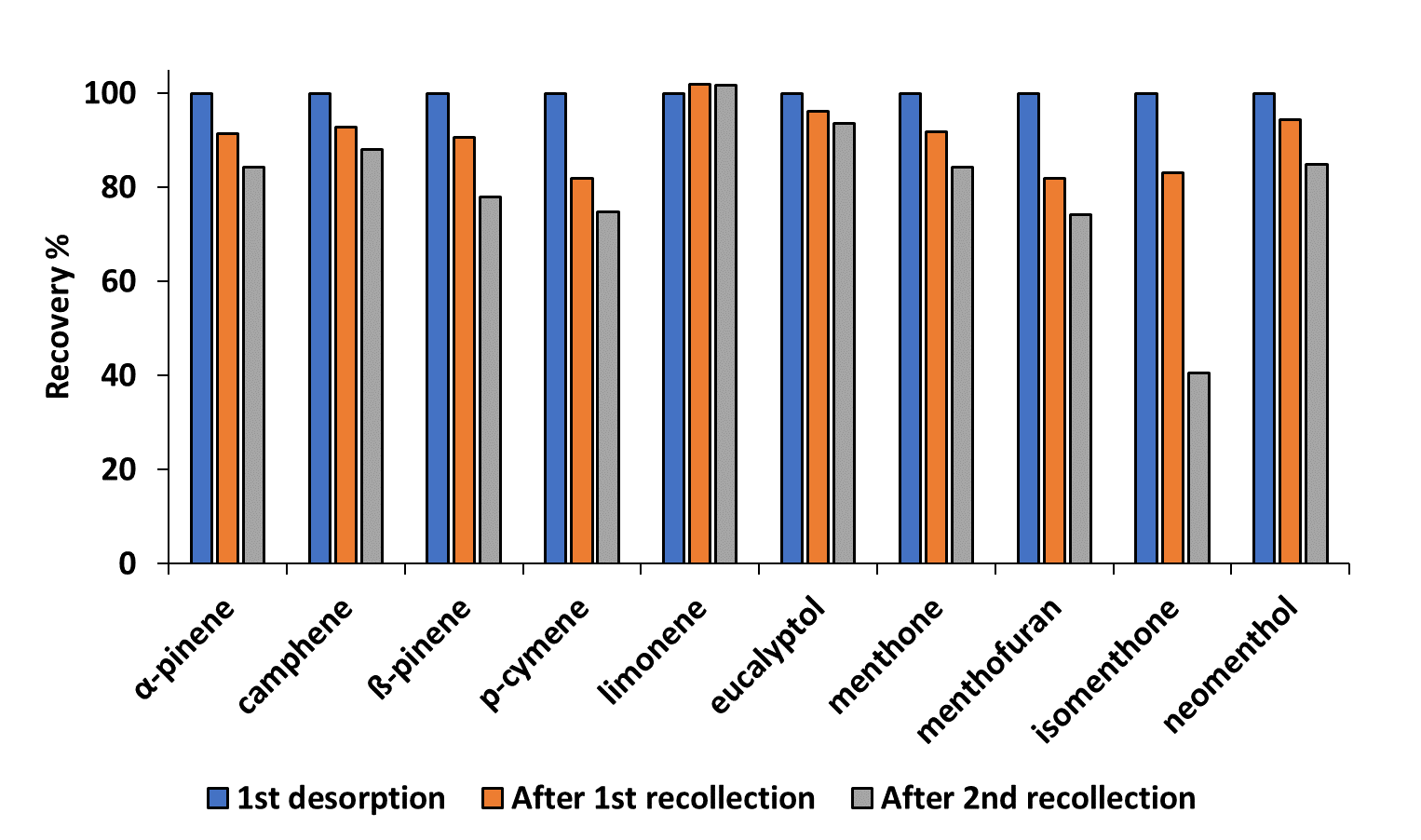
**

***Figure S4.*** *Recovery (expressed in %) of the 10 target analytes after 2 recollection cycles.*

**
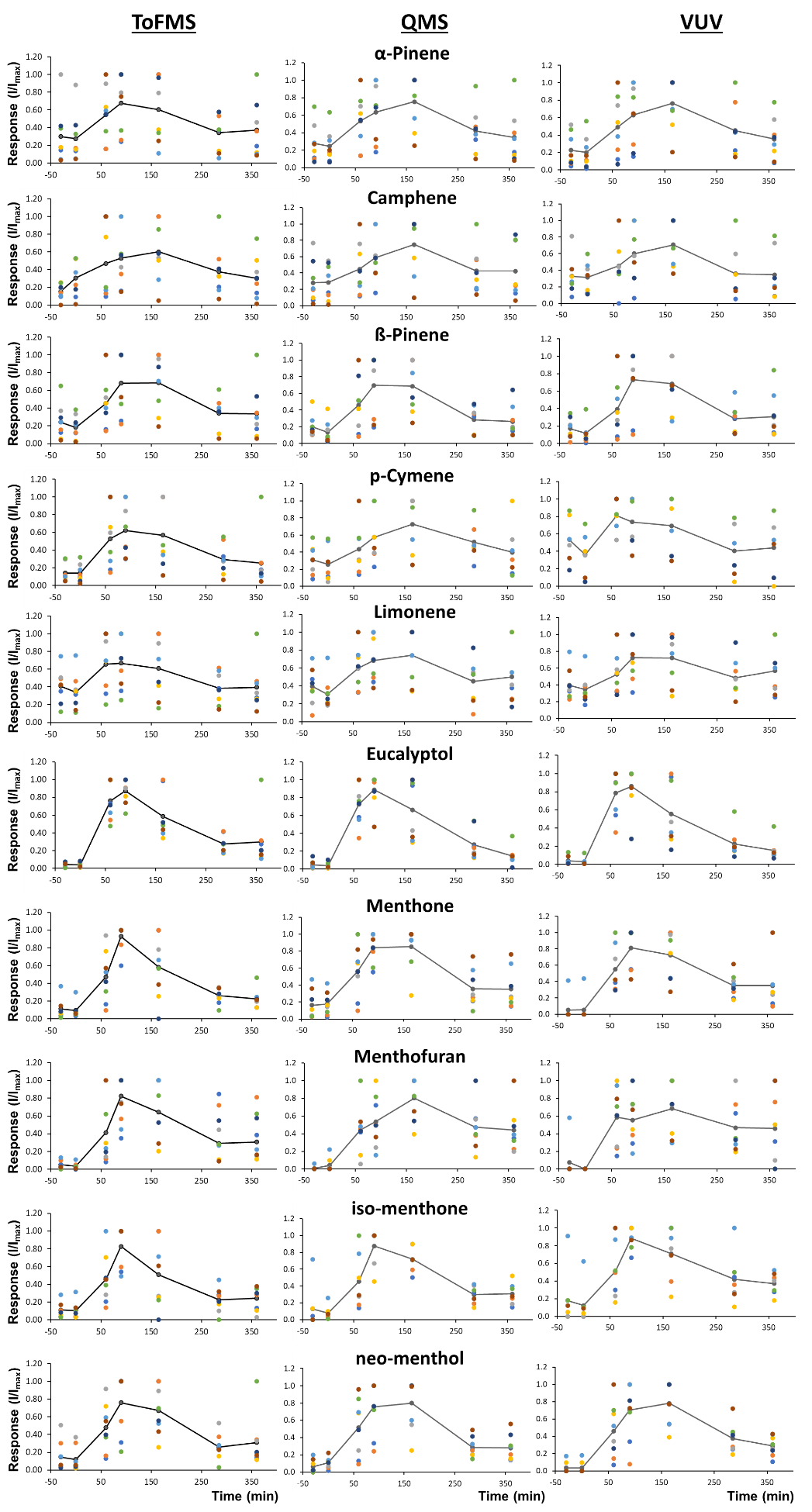
**

***Figure S5.*** *Washout curve of the peppermint metabolites for the 10 participants using the TD-GC×GC-ToFMS (left), and the TD-GC×GC-QMS/VUV (QMS, middle; VUV, right) platforms.*

**
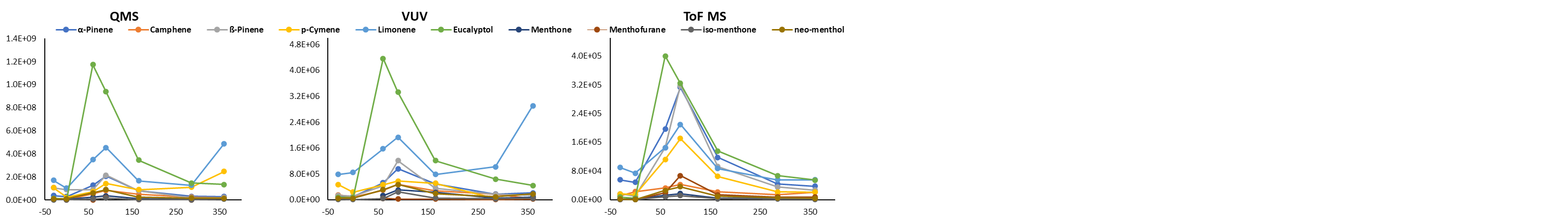
**

**Figure S6**. W*ashout curve of a representative exhaled breath sample using the TD-GC×GC-QMS/VUV (QMS, left; VUV, middle), and the TD-GC×GC-ToFMS (ToFMS, right) platforms.*


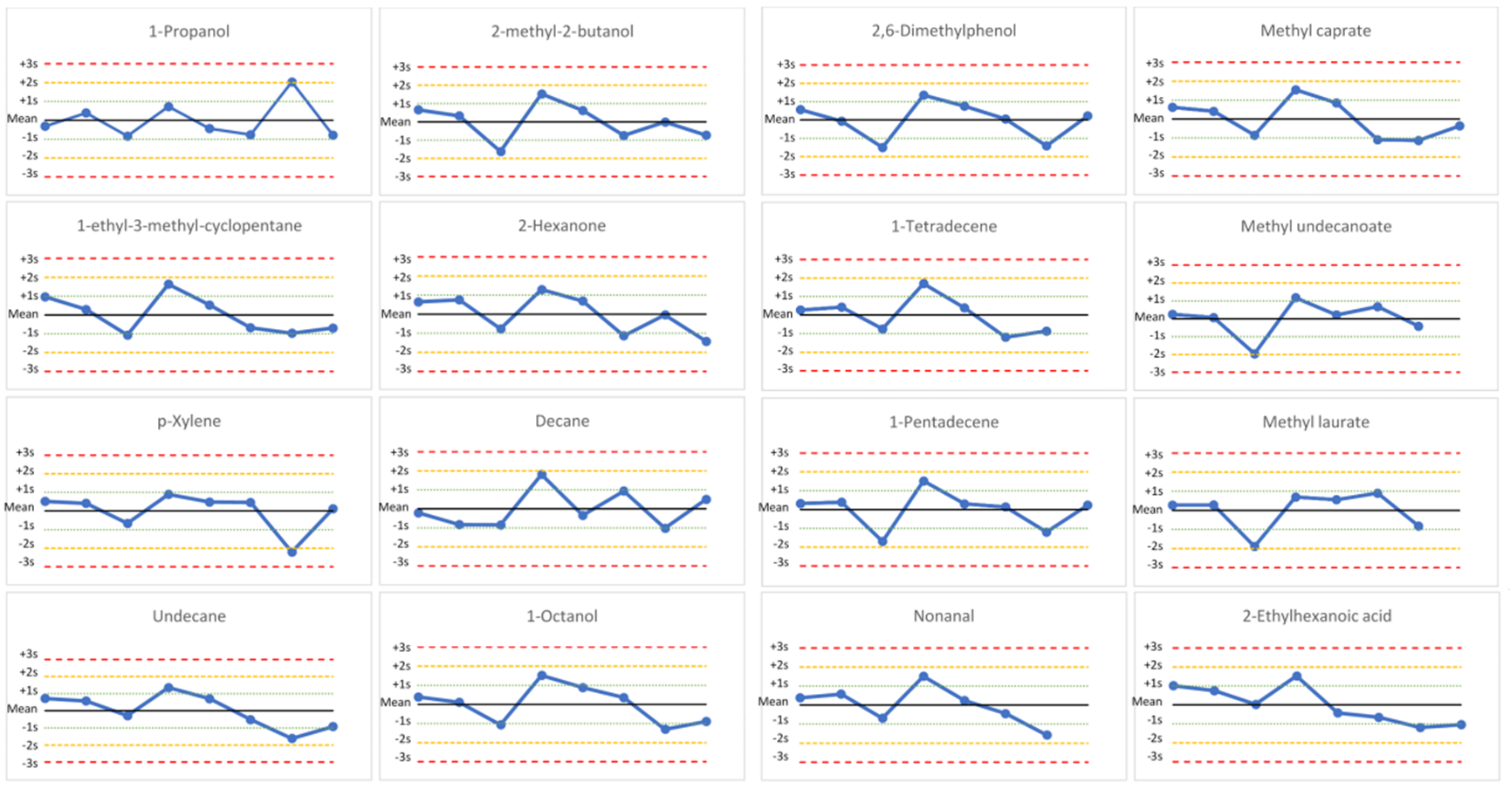


**Figure S7**. QC chart of a mixture of standards (16 compounds) injected weekly on platform 1.

***Table S1****. Coefficient of regression (R^2^) of the washout curves obtained between 90 or 165 min and 360 min for the three different detectors (i.e., QMS, VUV , and ToFMS).*

|  | **165-360 min** | | |
| --- | --- | --- | --- |
| **R^2^** | **QMS** | **VUV** | **ToFMS** |
| **alpha-pinene** | 0.996 | 0.998 | 0.891 |
| **camphene** | 0.926 | 0.942 | 0.892 |
| **beta-pinene** | 0.926 | 0.942 | 0.990 |
| **p-cymene** | 1.000 | 0.900 | 0.992 |
| **limonene** | 0.772 | 0.579 | 0.652 |
| **eucalyptol** | 0.985 | 1.000 | 0.972 |
| **menthone** | 0.920 | 0.916 | 0.980 |
| **menthofurane** | 0.979 | 0.935 | 0.949 |
| **isomenthone** | 0.945 | 0.980 | 0.960 |
| **neomenthol** | 0.926 | 0.995 | 0.958 |
